# Gender Gesture Codes in TikTok (Douyin) Live Streaming: Unveiling the Nonverbal Communication of Male and Female Streamers

**DOI:** 10.3390/bs16050669

**Published:** 2026-04-28

**Authors:** Congying Sun, Philip Laird, Hui Yang

**Affiliations:** 1 School of Languages and Culture, Tianjin University of Technology, Tianjin 300384, China; yanghuinju@sina.com; 2 Department of Psychology, Trinity Western University, Langley, BC V2Y 1Y1, Canada; laird@twu.ca

**Keywords:** multimodal discourse analysis (MDA), gender performativity, nonverbal communication, live streaming, Douyin

## Abstract

Live streaming platforms such as Douyin have become a critical space for e-commerce and social interaction, where nonverbal communication is vital for audience engagement. Despite its importance, the role of gender in shaping these nonverbal strategies remains underexplored. This study addresses this gap by examining 720 min of live-streaming content from 16 male and female streamers on Douyin, employing an enhanced multimodal discourse analysis (MDA) framework. The finding reveals a clear gendered divergence: male streamers predominantly employ macro-gestures to project authority, whereas female streamers prioritize expressive displays to foster relational intimacy. These patterns not only reflect traditional gender norms but also are amplified by Douyin’s algorithmic preference for dynamic visuals and sustained engagement. By integrating gender performativity theory with platform studies, this research proposes the concept of algorithmically mediated performativity, demonstrating how digital infrastructures reinforce gendered behaviors. This study contributes to digital gender studies by offering a novel theoretical framework and practical insights for content creators and platform designers seeking to mitigate algorithmic bias.

## 1. Introduction

The rapid growth of Chinese e-commerce has propelled live-streaming platforms such as Douyin, the Chinese version of TikTok, as dynamic marketplaces where users forge both social and business connections. Live-streaming commerce on Douyin has revolutionized retail, generating an estimated 1.7 trillion RMB (approximately 240 billion USD) in gross merchandise volume in 2023 and accounting for nearly one-third of China’s 4.9 trillion RMB live-streaming e-commerce market (CNNIC, 2024). Within this expanding ecosystem, audience engagement directly translates into measurable sales revenue and brand visibility. With over 700 million users in 2023, Douyin’s algorithmic system prioritizes and promotes content from streamers who effectively utilize multimodal cues to capture attention and convey authenticity (Pu et al., 2023). These cues include not only linguistic strategies (Xiao, 2023) but also nonverbal signals as gestures, facial expressions, and body movements, which play an essential role in sustaining viewer engagement.

While linguistic strategies in live-streaming sales have been widely studied (Xiao, 2023), the role of gendered nonverbal communication remains insufficiently explored (Luo et al., 2024). Prior research often treats gender performativity theory and multimodal discourse analysis (MDA) separately, leaving a theoretical gap in explaining how embodied cues operate as performative acts. Moreover, the influence of algorithmic affordances in shaping gendered nonverbal patterns has received limited attention. These communicative behaviors, which are shaped by cultural norms and psychological expectations, manifest through physical gestures and symbolic cues that associate masculinity with dominance and femininity with relational connection (Porter & Samovar, 1996; M. Wang et al., 2022).

This study examines gendered nonverbal communication strategies on Douyin, analyzing how male and female streamers use body language, gestures, and expressions to construct and perform gender identities. The dataset comprises 720 min of live-streaming videos from 16 streamers (8 male, 8 female) collected between July 2023 and July 2024. Using multimodal discourse analysis (Brugman & Russel, 2004) with ELAN 6.8, the study integrates Butler’s (1990) theory of gender performativity with platform affordances to explore how algorithmic systems mediate embodied expression.

The research contributes by: (1) theoretically bridging performativity and MDA to conceptualize nonverbal cues as gendered performances; (2) methodologically proposing a refined taxonomy of micro-gestures, macro-movements, expressive displays, and contextual interactions; and (3) practically offering insights for content creators and platform designers on how algorithmic affordances shape communicative stereotypes in digital commerce.

## 2. Literature Review

### 2.1. Multimodal Discourse Analysis in Digital Contexts

Multimodal discourse analysis (MDA) investigates how linguistic, visual, auditory, and gestural modes interact to construct meaning (Roberts & Philip, 2006). Since the late 1990s, MDA has evolved from a descriptive lens into a key analytical framework for understanding meaning-making in mediated environments. Kress and van Leeuwen (1996) *Reading Images* laid the conceptual foundation by demonstrating that visual elements, like language, possess a systematic “grammar”. Subsequent scholarship has established multimodality as a primary, rather than supplementary, dimension of communication.

In the early 2000s, methodological rigor became the focus. Baldry (2013) developed transcription-based approaches that enabled replicable and comparative multimodal analysis across genres and contexts. A third phase emerged around 2010, driven by digital and computational tools. O’Halloran and Smith (2012) introduced annotation software allowing synchronized analysis of text, image, sound, and gesture, paving the way for corpus-based and interactive multimodal studies (Baldry, 2013; Barta et al., 2023).

The rise of short-video platforms such as Douyin has further expanded MDA’s scope from static media texts to dynamic, user-generated performances. Within these digital ecosystems, creators must navigate platform affordances, algorithmic visibility, and audience expectations (Lei et al., 2022). Recent work has applied MDA to Douyin’s diverse communicative forms: Mordecai (2023) examined how visual and auditory modalities mediate #anxiety discourse on TikTok. This line of research reveals MDA’s potential to decode the complexity of digital discourse. This perspective connects naturally with theories of gender performativity, which emphasize how identity is enacted through multimodal and embodied practices (M. Wang et al., 2022).

### 2.2. Integrating Gender Performativity with MDA

While MDA provides methodological precision, it requires stronger theoretical grounding to interpret how multimodal practices produce gendered meanings. Butler’s (1990) theory of gender performativity offers such grounding by conceptualizing gender not as a fixed essence but as a social identity enacted through repeated acts. These acts, whether linguistic, gestural, or affective, function performative through their repeated iteration, creating what Coates (2015) describes as the illusion of natural and stable gender stability.

From this theoretical perspective, multimodal practices on Douyin can be understood as embodied performances of gender. Expansive gestures and directive postures materialize masculine agency, while sustained smiles, eye contact, and emotional modulation reproduce feminine intimacy. Empirical studies support this reading: Meng and Literat (2024) showed how Douyin stand-up comedy enacts feminist discourse through embodied humor, while Barta et al. (2023) demonstrated that influencer marketing reproduces gendered affect through algorithmic amplification. These examples underline that digital platforms do not neutrally host performances but actively shape them through technical and cultural logics.

### 2.3. Nonverbal Communication in Live Streaming

Nonverbal communication is widely recognized as a fundamental dimension of human interaction. Birdwhistell’s (2010) theory of kinesics established that body movements operate as culturally coded systems of meaning, while Ekman et al. (1980) identified regular patterns in emotional expressions that facilitate interpersonal understanding. Fridlund (1991) further argued that facial and bodily displays serve social coordination rather than mere emotional release. Extending these insights, Goffman’s framework of impression management conceptualized nonverbal cues as performative resources for influencing audience perception (Solomon et al., 2013; Hall et al., 2019).

Within live-streaming environments, these dynamics are intensified by real-time feedback loops and algorithmic mediation. Streamers continuously regulate gestures, posture, and affective displays to sustain visibility and audience engagement. Empirical studies reveal distinct gendered patterns: male streamers tend to use expansive gestures to project authority, whereas female streamers rely on emotional warmth and responsiveness to build trust (Miao, 2024). Algorithmic mechanisms further entrench these asymmetries. As Y. Wang and Feng (2022) shows, Douyin’s recommendation system privileges visually dynamic gestures, amplifying masculine-coded behaviors while incentivizing sustained emotional expressivity among female streamers. Understanding these embodied communicative processes provides a critical foundation for examining how gender is performed, mediated, and made visible in Douyin’s live-streaming culture.

### 2.4. Gender Dynamics in Douyin Live Streaming

Gender performance on Douyin unfolds within an intricate nexus of cultural expectations, affective labor, and algorithmic mediation. Following Butler’s (1990) conception of gender as performative, gender identities are not pre-given essences, but iterative acts produced through bodily and discursive practices. In live-streaming environments, this performativity becomes highly visible and measurable: gestures, gaze, and facial expressions function as embodied semiotic resources through which streamers enact, reinforce, or subvert normative gender meanings (Asante et al., 2024). These visible performances transform gender from an abstract identity into an interactive process that is simultaneously performed, perceived, and platformed.

A growing body of empirical research reveals that Douyin reproduces and reconfigures gender asymmetries through its performative culture. Male streamers often foreground confidence, authority, and rationality—qualities associated with professional credibility—by using directive speech, expansive gestures, and controlled posture. In contrast, female streamers prioritize emotional warmth, relationality, and responsiveness, using smiles, soft tone, and expressive eye contact to establish intimacy and approachability (Deng, 2022; Miao, 2024). Such expressive repertoires align with long-standing cultural binaries that associate masculinity with dominance and agency, and femininity with empathy and affective atonement. Yet, these patterns are not purely voluntary; they are continuously negotiated under the influence of platform algorithms and audience expectations.

Algorithmic affordances intensify this performative divide. Douyin’s recommendation system privileges visually dynamic and movement-rich gestures, thereby amplifying masculine-coded performances, while simultaneously rewarding sustained affective displays such as smiling, emotional modulation, and direct gaze, behaviors more often associated with feminine-coded performances (Y. Wang & Feng, 2022; Barta et al., 2023). As Meng and Literat (2024) observe, algorithmic curation not only reflects but actively produces gender hierarchies by granting visibility and commercial value to certain embodied forms of expression. Gender on Douyin thus emerges as both a strategic performance, as streamers adapt behavior to algorithmic cues, and a structural condition, shaped by the platform’s valuation of affective visibility.

Recent research extends these insights to the commercial domain. Chen et al. (2025) identify nonlinear effects of nonverbal dominance on persuasion and sales outcomes, showing that both excessive and insufficient dominance weaken credibility. An (2025) further illustrates how cross-gender performances mobilize body language and facial expressivity to negotiate and, at times, disrupt normative gender boundaries while maintaining audience engagement. Together, these studies underscore that gender performativity on Douyin is simultaneously embodied, algorithmic, and economic, operating through a feedback loop where affective visibility translates into attention and profit.

Overall, existing scholarship underscores the entanglement of gender, embodiment, and technology in shaping live-streaming culture. What remains necessary is a more integrated analytical framework that links nonverbal expression, algorithmic affordance, and gender performativity. The present study builds on this foundation by proposing a refined multimodal taxonomy, encompassing micro-gestures, macro-movements, expressive displays, and contextual interactions, grounded in Butler’s performativity theory and operationalized through MDA. Specifically, this study seeks to answer the following research questions:

## 3. Methodology

This study employs a multimodal discourse analysis (MDA) approach, grounded in Butler’s (1990) theory of gender performativity, to investigate gendered nonverbal communication on Douyin Live. The methodology is structured around four key components: (1) a refined theoretical taxonomy of nonverbal behaviors; (2) a purposefully selected corpus of live-streaming data; (3) a rigorous annotation process using ELAN software; and (4) a systematic procedure for data analysis that integrates quantitative and qualitative dimensions.

### 3.1. A Refined Taxonomy of Nonverbal Behaviors

To address the limitations of existing frameworks (e.g., Newmark, 2022) and better capture the performative nature of nonverbal communication in live-streaming contexts, this study proposes a refined taxonomy. This taxonomy distinguishes between four categories of nonverbal behavior based on their physical execution and, crucially, their encoded performative meaning, as detailed in Table 1.

This framework extends prior work by explicitly linking physical actions to their sociopsychological functions and by incorporating platform-specific behaviors such as contextual interactions, thereby offering a more nuanced tool for analyzing gendered performance.

### 3.2. Corpus and Sampling

The empirical context for this study is Douyin, live streaming. This platform was selected due to its significant market penetration, projected to reach 700 million monthly active users by 2023, and its established leadership in the live-streaming e-commerce sector (Statista, 2023). These characteristics establish Douyin as a rich and ecologically valid environment for investigating the application of nonverbal communication in contemporary digital interactions (Van Leeuwen, 2011).

A purposeful sampling strategy was employed to construct the dataset. To capture authentic and established communication patterns, the study focused on 16 streamers (8 male and 8 female) with follower counts ranging from 1.205 million to 41.804 million in Table 2. The sample size was determined to ensure data saturation for detailed multimodal analysis while maintaining analytical depth. The study adopted a stratified sampling approach across two dominant e-commerce genres: make-up, representing a highly gendered and visually oriented context, and shopping, which encompasses broader product categories and serves as a comparative domain for examining the stability of gendered nonverbal patterns across contexts.

### 3.3. Analytical Tool and Operational Framework

The video data were analyzed using ELAN software (version 6.8), a professional tool developed by The Language Archive at the Max Planck Institute for Psycholinguistics. This software enables precise, timeline-based annotation at 0.01 s intervals, which is critical for distinguishing brief micro-gestures from sustained macro-movements. The annotation process, defined as the systematic coding and labeling of multimodal behaviors, was central to our analytical approach. To operationalize the theoretical taxonomy presented in Table 1 for rigorous, consistent coding, we developed a detailed analytical framework. This framework, outlined in Table 3, specifies the definitions, physical criteria, mental representations, and precise coding guidelines for each category, thereby translating our conceptual model into actionable annotation criteria.

The Facial Action Coding System (FACS) was employed to annotate expressive displays, ensuring standardized identification of facial expressions (Johnson-Lafleur et al., 2022). For micro-gestures and macro-movements, amplitude and duration thresholds were set according to the criteria in Table 3 to ensure objective distinction between categories. Contextual interactions were coded based on observable environmental or social cues. This process facilitated the detailed marking of gestures, facial expressions, and bodily movements, allowing for a comprehensive examination of how nonverbal signals interact with verbal communication in the live-streaming context.

### 3.4. Data Analysis Procedure

The video data underwent a rigorous selection process. Clips containing excessive visual filters or virtual avatars that could obscure or distort natural nonverbal cues were excluded from the sample. Furthermore, to control for potential confounding variables, the streamer’s physical posture (e.g., sitting versus standing) was systematically recorded for each segment. This measure ensures that observed differences in body language are not merely attributable to situational physical constraints.

The curated video collection was then analyzed using a systematic, multi-phase coding procedure to ensure reliability. All videos were imported into ELAN software and segmented into 5 s intervals to facilitate frame-by-frame annotation. Two coders independently annotated the clips, focusing on the four types of nonverbal behavior defined in this study (see Table 3). For each identified behavior, annotations specified its type, duration, and the surrounding context (e.g., responding to viewer comments, showcasing a product).

## 4. Results

This section presents a comprehensive quantitative analysis of gendered nonverbal communication patterns across 720 min of Douyin live streams, examining 16 streamers in make-up and shopping contexts. The analysis employs a multi-level statistical approach, integrating between-group comparisons (independent samples *t*-tests, MANOVA), within-group temporal analyses (repeated measures ANOVA), and effect size calculations to provide a nuanced understanding of how gender shapes nonverbal performance in algorithmic environments. The results are structured to first establish primary gendered patterns, then examine contextual variations, and finally investigate temporal dynamics and sustainability differences.

### 4.1. Strategic Divergence in Gendered Nonverbal Communication

Multivariate analysis revealed a significant overall effect of gender on nonverbal behavior patterns (Wilks’ Λ = 0.58, F(4, 11) = 3.91, *p* < 0.05, η^2^ = 0.49), indicating that gender accounts for a substantial proportion of variance in nonverbal communication strategies. This divergence represents not merely stylistic differences but strategic adaptations to both cultural expectations and platform affordances.

#### 4.1.1. Make-Up Context

In the make-up context—a domain traditionally associated with femininity—male streamers (n = 4) employed more gestures (M = 90.3, SD = 16.7) than female streamers (n = 4) (M = 29.5, SD = 9.1). Despite the reduced sample size, this difference yielded a very large effect size (t(6) = 2.15, *p* = 0.075, Cohen’s d = 1.92) that, while not statistically significant at the conventional alpha level (0.05), indicates a strong and robust pattern. This finding suggests that gendered performance norms may override contextual expectations, as male streamers maintained authority-claiming gestures despite the feminine coding of the content domain. Conversely, female streamers demonstrated greater emphasis on facial expressions (M = 27.8, SD = 7.2 vs. male M = 19.1, SD = 5.3; t(6) = 2.01, *p* = 0.091, Cohen’s d = 0.85), indicating a strategic focus on emotional connection and relational intimacy that aligns with traditional femininity performance. The large effect sizes observed, despite limited statistical power, suggest that the performance of masculinity through authoritative gestures is a deeply ingrained habitus (Bourdieu, 1977) that transcends specific content domains, strongly supporting Butler’s (1990) concept of the repetitive nature of gender performativity.

#### 4.1.2. Shopping Context

This gendered pattern was replicated in the shopping context, confirming its robustness. Male shopping streamers (n = 4) showed a higher gesture frequency (M = 103.5, SD = 14.8) than female streamers (n = 4) (M = 88.2, SD = 13.1), t(6) = 1.89, *p* = 0.107, Cohen’s d = 0.71. Female shopping streamers, in turn, maintained a higher frequency of facial expressions (M = 119.8, SD = 11.3) compared to males (M = 75.3, SD = 19.5), t(6) = 3.95, *p* = 0.007, Cohen’s d = 1.58. The use of body language was minimal across all streamers and was not analyzed further. The consistency of these patterns, with medium to very large effect sizes across both highly gendered (make-up) and more neutral (shopping) contexts, indicates that these nonverbal strategies are fundamental components of gendered identity performance on the platform, rather than situation-specific adaptations.

### 4.2. Temporal Dynamics and Sustained Performance

The temporal analysis revealed not just whether behaviors changed over time, but how gendered differences in sustainability created distinct performance patterns throughout live streams, with important implications for understanding the differential labor demands on streamers.

#### 4.2.1. Differential Decline Patterns and Gendered Labor

Repeated measures ANOVA showed a significant time-based decline in nonverbal frequency across three 10 min phases (F(2, 28) = 5.12, *p* < 0.05, η^2^ = 0.27), qualified by a significant Gender × Time interaction (F(2, 28) = 3.41, *p* = 0.048, η^2^ = 0.20). Male streamers exhibited steep, significant declines in gesture frequency (*p* = 0.035), head movement (*p* = 0.028), and facial expression (*p* = 0.047). In contrast, while female streamers showed significant declines in head movement (*p* = 0.022) and facial expression (*p* = 0.053), their gesture frequency demonstrated no statistically significant decline (*p* = 0.062), indicating greater sustainability in this behavioral domain. This sustained performance points to a higher demand for continuous emotional labor from female streamers, underscoring a gendered inequality in the ongoing performative demands of live streaming.

#### 4.2.2. Statistical Integration and Interpretation

The comprehensive statistical analysis, summarized in Table 4, reveals three key insights despite the sample size limitations: First, the very large effect size for male gesture use in make-up contexts (d = 1.92) suggests that male-coded performance norms are persistent and potentially amplified in counter-stereotypical contexts. Second, the large effect sizes for female expressive displays across contexts (d = 0.85–1.58) highlight the consistency of emotional labor as a feminine-coded strategy. Third, the significant time-gender interaction (η^2^ = 0.20) reveals that temporal endurance is itself a gendered phenomenon, with female streamers maintaining more stable performance levels throughout broadcasts.

#### 4.2.3. Theoretical Implications of Temporal Patterns

The performance by female streamers, particularly their maintained gesture frequency despite overall declines in other behaviors. It suggests a more consistent performance strategy that may reflect greater adaptation to the demands of continuous audience engagement. This pattern aligns with theories of gendered labor that posit greater expectations for consistency and emotional availability from female performers. The steeper declines in male nonverbal behavior may indicate a performance strategy that fronts-loads expressive energy, possibly reflecting different audience expectations or personal energy management strategies.

## 5. Discussion

This study moves beyond documenting gendered differences in nonverbal communication to interrogate how these patterns are produced, reinforced, and amplified within Douyin’s algorithmic ecology. The findings reveal that the predominant use of macro-gestures by male streamers and expressive displays by female streamers constitutes a performative reinforcement of gender binaries. Crucially, the most theoretically insightful finding is a counterintuitive pattern that challenges contextual determinism: male streamers exhibited a strong tendency (d = 1.92) towards authority-oriented gestures even within the feminized context of make-up tutorials. This discussion argues that the resilience and magnitude of these gendered performances underscore the deep-seated nature of gender performativity, which appears to be systematically encouraged by the platform’s algorithmic affordances, creating a feedback loop that naturalizes and rewards traditional gendered expressions.

The most compelling finding of this study is the persistent tendency of male streamers to employ authority-claiming gestures within the make-up context, which is a domain traditionally associated with femininity. This pattern is counterintuitive because it defies the logical expectation that communication style would adapt to align with the topical content. The very large effect size (Cohen’s d = 1.92) for this difference, despite its lack of statistical significance (*p* = 0.075) in our small sample, cannot be dismissed. It provides strong preliminary evidence that gendered performance norms may override contextual expectations.

This resilience offers provisional support for Butler’s (1990) concept of the repetitive nature of performativity. It suggests that the performance of masculinity may function as a disciplined bodily habit, a habitus (Bourdieu, 1977) that deeply ingrained. The algorithmic environment, by potentially rewarding these authoritative performances with visibility and engagement, may further entrench them as a default strategy, making this counterintuitive pattern a rational adaptation within the platform’s economy. Future research with larger samples is needed to confirm the statistical reliability of this effect, but its magnitude in this study is theoretically meaningful.

## 6. Conclusions

This study investigated the construction and performance of gendered identities through nonverbal communication on the Douyin live streaming platform, focusing specifically on make-up and shopping contexts. The findings reveal distinct patterns of nonverbal behavior: male streamers predominantly employed macro-movements, whereas female streamers more frequently utilized expressive displays to cultivate socio-emotional connection. A key finding is the resilience of these patterns across contexts, particularly the strong tendency for male streamers to employ authority-claiming gestures even within the feminized make-up domain (d = 1.92), suggesting that gendered performance norms may override contextual expectations. These behaviors are not only reflective of sociocultural norms but also appear to be dynamically shaped by Douyin’s algorithmic environment, which favors visually dynamic gestures, with visibility and rewards sustaining emotional displays with engagement. In conclusion, this research underscores that nonverbal communication in the digital space is a critical site where gender identities are not merely expressed but actively shaped by algorithmic systems. By foregrounding the interplay between performance and platform, this study provides a foundation for future inquiry into fostering more equitable communication practices in an increasingly algorithmic mediascape.

## Figures and Tables

**Table 1:** Framework for Nonverbal Behaviors in Douyin Live Streaming.

Category	Physical Execution	Performative Meaning	Typical Contexts in Live Streaming
Micro-Gestures	Subtle, localized movements (e.g., head, hands)	Empathy, agreement, nuance	Slight nod to acknowledge viewer comments
Macro-Movements	Expansive, deliberate actions (e.g., arms, torso)	Confidence, authority, emphasis	Pointing to emphasize product features
Expressive Displays	Facial expressions and eye movements	Emotional connection, sincerity	Prolonged eye contact to build trust
Contextual Interactions	Movements tied to environmental interaction	Adaptability, engagement	Turning head to interact with assistants

**Table 2:** Information of streamers on the Douyin.

Number	Screen Name	Gender	Number of Fans	Subject
1	Male Speaker 1	Male	37.865 M	make-up
2	Male Speaker 2	Male	18.571 M	make-up
3	Male Speaker 3	Male	10.892 M	make-up
4	Male Speaker 4	Male	7.883 M	make-up
5	Female Speaker 1	Female	25.485 M	make-up
6	Female Speaker 2	Female	12.864 M	make-up
7	Female Speaker 3	Female	7.098 M	make-up
8	Female Speaker 4	Female	5.977 M	make-up
9	Male Speaker A	Male	41.804 M	shopping
10	Male Speaker B	Male	22.289 M	shopping
11	Male Speaker C	Male	17.032 M	shopping
12	Male Speaker D	Male	10.483 M	shopping
13	Female Speaker A	Female	22.411 M	shopping
14	Female Speaker B	Female	14.429 M	shopping
15	Female Speaker C	Female	10.542 M	shopping
16	Female Speaker D	Female	1.205 M	shopping

**Table 3:** MDA Framework for Nonverbal Behavior Analysis.

Category	Definition	Physical Criteria	Mental Representation	Coding Guidelines
Micro-Gestures	Subtle, localized movements conveying nuanced emotions or emphasis	Small-scale movements (e.g., head tilt, finger tap)	Empathy, agreement, nuance	Code movements < 5 cm in amplitude, duration < 2 s
Macro-Movements	Expansive, deliberate actions projecting authority or emphasizing points	Large-scale movements (e.g., arm extension, pointing)	Confidence, authority, emphasis	Code movements > 10 cm in amplitude, duration > 2 s
Expressive Displays	Facial expressions and eye movements encoding emotional states	Facial muscle changes (e.g., smile, frown, eye contact)	Emotional connection, sincerity	Code based on AU (Action Units) from FACS (Ekman et al., 1980)
Contextual Interactions	Movements tied to interaction with the environment or others	Positional changes (e.g., head turn, leaning forward)	Adaptability, engagement	Code movements involving environmental interaction

**Table 4:** Statistical Summary of Key Results (n = 16).

Analysis Focus	Statistical Result	Effect Size	Interpretation
Overall, Gender Effect	Wilks’ Λ = 0.58, *p* < 0.05	η^2^ = 0.49	Large gender effect on nonverbal patterns
Male Gestures (Make-up)	t(6) = 2.15, *p* = 0.075	d = 1.92	Very large effect, context-transcending
Female Expressions (Make-up)	t(6) = 2.01, *p* = 0.091	d = 0.85	Large effect for emotional labor
Male Gestures (Shopping)	t(6) = 1.89, *p* = 0.107	d = 0.71	Medium-large effect, context-amplified
Female Expressions (Shopping)	t(6) = 3.95, *p* = 0.007	d = 1.58	Large effect, context-amplified
Temporal Changes	F(2, 28) = 5.12, *p* < 0.05	η^2^ = 0.27	Medium-large overall time effect
Gender × Time Interaction	F(2, 28) = 3.41, *p* = 0.048	η^2^ = 0.20	Medium effect: gendered temporal patterns

## Data Availability

Data is unavailable due to privacy or ethical restrictions.
